# Higher basal tryptase, asthma and loss of consciousness in anaphylaxis are associated with biphasic reactions

**DOI:** 10.1002/clt2.12166

**Published:** 2022-06-15

**Authors:** Terence Langlois, Pascale Nicaise‐Roland, Camille Taillé, Patrick Natta, Bruno Crestani, Sylvie Chollet‐Martin, Luc de Chaisemartin, Catherine Neukirch

**Affiliations:** ^1^ Service de pneumologie A APHP Hôpital Bichat Paris France; ^2^ Université Paris‐Saclay Paris France; ^3^ Service d’Immunologie APHP Hôpital Bichat Paris France; ^4^ Université de Paris Cité Inserm 1152 Paris France; ^5^ Université Paris‐Saclay Inserm Inflammation Microbiome et Immunosurveillance Châtenay‐Malabry France

To the Editor

Anaphylaxis is the most severe form of immediate hypersensitivity, requiring fast and appropriate medical management.^1^ After a first hypersensitivity phase, a biphasic reaction occurs in 4%–6% of patients^2^ without any elicitor re‐exposure. Biphasic reactions are known to be more severe than monophasic anaphylaxis.^3^ Therefore, close monitoring is recommended up to 12 h after an anaphylactic reaction. Identification of patients at risk for a biphasic reaction is important to improve patients care.

In a population of adult patients (*n* = 237) referred for anaphylaxis (according to the 2020 World Allergy Organization criteria)^1^ to our reference center for allergy in a tertiary‐care university hospital, from January 2017 to May 2020, we retrospectively compared patients with a monophasic anaphylaxis or a biphasic anaphylaxis. Patients with a diagnosis of mast cell disease were excluded. Among the 237 patients, 13 patients had a biphasic reaction (5.5%). Characteristics of monophasic and biphasic patients are listed in Table 1. The mean delay between the first and second reaction was 8 h (range 1–48). The odds of a biphasic reaction was increased with asthma (*n* = 6/13, 46.1%; vs. 38/224, 16.9%; odds ratio = 4 [95% CI 1.05–14.81], *p* = 0.02) and loss of consciousness during anaphylaxis (*n* = 13/13, 100% of biphasic patients vs. 132/224, 58.9% of monophasic patients, *p* = 0.008), as compared with monophasic anaphylaxis (Table 1). Basal tryptase levels were significantly higher with biphasic than monophasic anaphylaxis (median: 6.1 µg/l, vs. 4.2 µg/l respectively, *p* = 0.009) (Table 1). For asthma patients, basal tryptase level was significantly higher for those with biphasic than monophasic anaphylaxis (median: 5.3 µg/l, *n* = 6 vs. 3.7 µg/l, *n* = 38, *p* = 0.015).

**TABLE 1:** Clinical characteristics, features and elicitors for monophasic and biphasic anaphylaxis

	Monophasic anaphylaxis *n* = 224	Biphasic anaphylaxis *n* = 13	*p*‐value
**Basal tryptase,** median (SD) μg/L	**4,2 (3,1)**	**6,1 (5,4)**	***p* = 0.009**
Demography and clinical characteristics			
Men	87 (38.8%)	5 (38.5%)	*p* = 1
Age, mean (SD)	49.2 (16.7)	49.8 (17.4)	*p* = 1
**Asthma**	**38 (16.9%)**	**6 (46.1%)**	***p* = 0.021** ^**a**^
Inhaled steroids	19/38 (50%)	2/6 (33%)	*p* = 0.26
Allergic rhino‐conjunctivitis	77 (34.4%)	7 (53.8%)	*p* = 0.24
Contact eczema	41 (18.3%)	3 (23.1%)	*p* = 0.72
Chronic urticaria	5 (2.2%)	0	*p* = 1
Atopic dermatitis	16 (7.1%)	1 (7.7%)	*p* = 1
History of hymenoptera hypersensitivity	4 (1.8%)	0	*p* = 1
History of drug hypersensitivity	13 (5.8%)	0	*p* = 1
History of food hypersensitivity	34 (15.2%)	3 (23.1%)	*p* = 0.44
Cardiovascular disease	73 (32.6%)	4 (30.8%)	*p* = 1
Diabetes	14 (6.2%)	1 (7.7%)	*p* = 0.58
Dysthyroidism	18 (8.0%)	1 (7.7%)	*p* = 1
Renal failure	1 (0.4%)	0	*p* = 1
Smoking	85 (37.9%)	7 (53.8%)	*p* = 0.26
Beta‐blocker treatment	26 (11.6%)	0	*p* = 0.37
PPI treatment	44 (19.6%)	2 (15.4%)	*p* = 1
Anaphylaxis symptoms and treatment			
Severity grade II	112 (50%)	6 (46.1%)	*p* = 1
Severity grade III + IV	112 (50%)	7 (53.8%)	*p* = 1
Skin signs	182 (81.2%)	11 (84.6%)	*p* = 1
Respiratory signs	118 (52.7%)	7 (53.8%)	*p* = 0.98
Digestive signs	39 (17.4%)	5 (38.5%)	*p* = 0.14
**Loss of consciousness**	**132 (58.9%)**	**13 (100%)**	***p* = 0.008** ^**b**^
Epinephrine treatment engaged	99 (44.2%)	9 (69.2%)	*p* = 0.39
Elicitors			
Antibiotics	34 (15.2%)	2 (15.4%)	*p* = 1
Neuro‐muscular blocking agents	57 (25.4%)	4 (30.8%)	*P* = 0.74
Iodinated contrast media	11 (4.9%)	0	*p* = 1
NSAIDs/aspirin	15 (6.7%)	0	*p* = 1
Paracetamol	1 (0.4%)	0	*p* = 1
Others drugs	1 (0.4%)	0	*p* = 1
Hymenoptera venom	50 (22.3%)	6 (46.2%)	*p* = 0.39
Food	3 (1.3%)	1 (7.6%)	*p* = 0.22
Unknown elicitor	52 (23.2%)	0	*p* = 0.08

^a^ OR = 4 CI [1,05; 14,81].

^b^ OR = Infini [CI95].

This is the first study to suggest that basal tryptase level was higher with biphasic than monophasic anaphylaxis and we know that higher tryptase level is associated with severity of anaphylaxis.^1^, ^4^ This association needs to be confirmed in futures studies to conclude that higher basal tryptase could be a risk factor for severe anaphylaxis like biphasic reactions. The increased proportion of asthma patients in the biphasic group might be an explanation. The association between severe anaphylaxis and biphasic reactions was recently investigated in a large cohort (8736 patients with monophasic and 435 with biphasic anaphylaxis).^3^ In this study, Kraft et al. found no significant difference in mean basal tryptase level between monophasic and biphasic anaphylaxis. However, the authors included patients with systemic mastocytosis in their analysis, which could perhaps mask a difference in basal tryptase level and explain the discrepancy with our results. We found the loss of consciousness was more frequent in patients with a biphasic anaphylaxis, suggesting a more severe reaction. Gastrointestinal symptoms, skin symptoms, cardiac symptoms, respiratory arrest, and chronic urticaria were associated with the occurrence of biphasic reaction in the Kraft et al. study.^3^ While we did not find such associations, possibly due to the limited number of biphasic patients in our study, we show for the first time a link between asthma comorbidity and biphasic anaphylaxis. The proportion of asthma patients in our cohort is similar to what is observed in other cohorts (18.5% in the current series vs. 22.5% in a large anaphylaxis registry).^5^ Asthma is known to increase the severity of anaphylaxis^1^ and severe asthma was recently found associated with elevated basal tryptase level (independently of type 2 inflammation).^6^

In conclusion, a diagnosis of asthma and loss of consciousness during the first phase of anaphylaxis could be associated with a biphasic reaction. These results advocate for prolonged monitoring of these patients during their care. Higher basal tryptase was linked to biphasic reactions. This finding could help anticipate biphasic reactions for patients with a history of immediate hypersensitivity and better understand the mechanisms of such reactions in future studies.

## CONFLICT OF INTEREST

The authors declare that they do not have conflict of interests related to the contents of this article.

## AUTHOR CONTRIBUTIONS

**Terence Langlois:** Conceptualization (Lead); Data curation (Lead); Formal analysis (Lead); Investigation (Lead); Methodology (Lead); Project administration (Equal); Resources (Equal); Supervision (Equal); Validation (Equal); Visualization (Lead); Writing – original draft (Lead); Writing – review & editing (Equal). **Pascale Nicaise‐Roland:** Investigation (Equal); Methodology (Equal); Resources (Equal); Supervision (Equal); Visualization (Equal); Writing – original draft (Supporting); Writing – review & editing (Supporting). **Camille Taillé:** Investigation (Equal); Methodology (Equal); Project administration (Equal); Resources (Equal); Supervision (Lead); Validation (Lead); Visualization (Equal); Writing – original draft (Equal); Writing – review & editing (Equal). **Patrick Natta:** Investigation (Equal); Resources (Equal); Validation (Equal); Visualization (Supporting); Writing – original draft (Supporting). **Bruno Crestani:** Investigation (Equal); Methodology (Equal); Project administration (Equal); Resources (Equal); Supervision (Equal); Validation (Lead); Visualization (Equal); Writing – original draft (Equal); Writing – review & editing (Equal). **Sylvie Chollet‐Martin:** Investigation (Equal); Methodology (Equal); Resources (Equal); Supervision (Equal); Validation (Equal); Visualization (Equal); Writing – original draft (Supporting); Writing – review & editing (Supporting). **Luc de Chaisemartin:** Conceptualization (Supporting); Data curation (Supporting); Formal analysis (Supporting); Investigation (Equal); Methodology (Equal); Project administration (Supporting); Resources (Equal); Supervision (Equal); Validation (Equal); Visualization (Equal); Writing – original draft (Lead); Writing – review & editing (Equal). **Catherine Neukirch:** Conceptualization (Lead); Data curation (Equal); Formal analysis (Equal); Investigation (Lead); Methodology (Lead); Project administration (Lead); Resources (Lead); Software (Supporting); Supervision (Lead); Validation (Lead); Visualization (Lead); Writing – original draft (Lead); Writing – review & editing (Lead).

## Data Availability

The data that support the findings of this study are available on request from the corresponding author. The data are not publicly available due to privacy or ethical restrictions.
